# No Association Between MicroRNA-608 rs4919510 G>C Polymorphism and Digestive System Cancers Susceptibility: A Meta-Analysis Based on 10,836 Individuals

**DOI:** 10.3389/fphys.2018.00705

**Published:** 2018-06-07

**Authors:** Xue-Feng Li, Ju-Kun Song, Jun-Wei Cai, Yu-Qin Zeng, Min Li, Jie Zhu, Yu-Ming Niu

**Affiliations:** ^1^Department of Endocrinology, Taihe Hospital, Hubei University of Medicine, Shiyan, China; ^2^Department of Oral and Maxillary Surgery, Gui Zhou Provincial People's Hospital, Guiyang, China; ^3^Trade Union, Taihe Hospital, Hubei University of Medicine, Shiyan, China; ^4^Department of Stomatology, Center for Evidence-Based Medicine, Taihe Hospital, Hubei University of Medicine, Shiyan, China

**Keywords:** microRNA-608, rs4919510, cancer, polymorphism, susceptibility

## Abstract

Previous epidemiologic studies have revealed a possible association between microRNA-608 rs4919510 G>C polymorphism and digestive system cancers (DSCs) risk, but the results were not consistent. We therefore performed an updated meta-analysis to explore the association between microRNA-608 rs4919510 G>C polymorphism and DSCs risk. Crude odds ratios (ORs) with 95% confidence intervals (CIs) were calculated to assess the relationship between the microRNA-608 rs4919510 G>C polymorphism and DSCs risk. Heterogeneity, cumulative analyses, sensitivity analyses, and publication bias were also conducted to examine the statistical power. Eight published articles with nine independent case-control studies involving 10,836 individuals were included in this meta-analysis. Overall, no significant association was found between microRNA-608 rs4919510 G>C polymorphism and DSCs risk in general populations. But some significant protective effects were observed in the subgroup of Caucasian population group in three genetic models (C vs. G: OR = 0.82, 95% CI, 0.68–0.99, *P* = 0.03, *I*^2^ = 0%; CC vs. GG: OR = 0.59, 95% CI = 0.36–0.97, *P* = 0.04, *I*^2^ = 0%; GC+CC vs. GG: OR = 0.61, 95% CI = 0.37–0.99, *P* = 0.05, *I*^2^ = 0%). In summary, current evidence indicates that the microRNA-608 rs4919510 G>C polymorphism maybe an important factor of DSCs susceptibility, especially in Caucasian population.

## Introduction

Digestive system cancers (DSCs), which comprise esophageal cancer (EC), gastric cancer (GC), hepatocellular cancer (HC), pancreatic cancer (PC) colorectal cancer (CRC) and other solid carcinoma, is one of the most common malignancies with increasing incidence and mortality worldwide (Torre et al., 2015). According to the recent published cancer statistics, there were approximately 310,440 new DSCs cases and 157,700 DSCs-related deaths in United States in 2017 (Siegel et al., 2017). The treatment and care of patients with DSCs result in a heavy economic and psychological burden to both society and the patient's family (Hsu et al., 2017; Jinjuvadia et al., 2017). Surgery, radiotherapy, chemotherapy and other treatments may cause extensive damage to patients' tissues and organs and may result in various complications that lead to local dysfunction and seriously decrease the quality of life of patients (Nederlof et al., 2016; Bosch et al., 2017; Motoyama et al., 2017). Regrettably, no clear explanation of the mechanism underlying DSCs development and the susceptibility of different patients exist. Some researches indicate that unhealthy life styles, cigarette and alcohol use, viral infection, local inflammation and stress can trigger DSCs development (Erren et al., 2016; Gao et al., 2017; Jarzynski et al., 2017; Jayasekara et al., 2018).

Evidences suggested that abnormal gene expression and an alteration of amino acid structure can lead to abnormal biologic activities that result in an imbalance of normal physiologic function and induce the formation of tumors. MicroRNAs were discovered in 1993 and were defined as small noncoding RNA molecules with approximately 21–25 nucleotides in length and a characteristic double-stranded structure (Lee et al., 1993; Ambros, 2004). MicroRNAs always originate from endogenous transcripts and bind to imperfect complementary sequences of the 3′-untranslated regions (3'-UTR) of target mRNAs to regulate their post-transcriptional repression (Bartel, 2004). Some studies indicated that abnormal microRNA expression could result in tumor occurrence (Valeri et al., 2010; Tessitore et al., 2014). MicroRNA-608 is a newly discovered microRNA, as reported by Wang et al., who indicated that the expression of microRNA-608 was significantly reduced in hepatocellular cancer and its expression levels were associated with tumor size, differentiation, clinical stage, and overall prognosis (Wang K. et al., 2016). Further, other researchers found that the expression of microRNA-608 was significantly down-regulated in glioma stem cells (GSCs). And the up-regulation of microRNA-608 expression would inhibit the proliferation, migration, and invasion of GSCs and promotes their apoptosis (Wang Z. et al., 2016).

Single nucleotide polymorphisms (SNPs) are the most common type of genetic variation in people. SNPs alter gene function and/or expression, consequently affecting downstream biologic pathways and increasing cancer risk (Martini et al., 2013; Niu et al., 2015). In microRNA-608, rs4919510 G>C is the most common locus and has attracted increasing attention. Ye et al. conducted the first case-control study in 2008 and did not find any significant association between the microRNA-608 rs4919510 G>C polymorphism and esophageal cancer (Ye et al., 2008). Recently, a published meta-analysis addressed the association between microRNA-608 rs4919510 G>C polymorphism and cancer risk (Liu et al., 2016), but the results were not consistent with subsequent studies, especially in DSCs. Therefore, we conducted the present meta-analysis to more precisely and comprehensively assess of the association between microRNA-608 rs4919510 G>C polymorphism and DSCs risk.

## Materials and methods

This present meta-analysis followed the Preferred Reporting Items for Systematic Reviews and Meta-Analyses (PRISMA) statement (Moher et al., 2009). All included data were collected from published studies, and no ethical issues were involved.

### Search strategy

Five electronic English databases (PubMed, Embase, Web of Science, CNKI and Wanfang) were searched for relevant studies that focused on the association between microRNA-608 rs4919510 G>C polymorphism and DSCs risk from inception up to January 1, 2018. Only studies that were written in English and Chinese were included. Moreover, the bibliographies of the collected studies and relevant reviews were retrospected to identify additional articles. The following search terms and strategy was used (e.g., in PubMed):

#1 microRNA 608

#2 microRNA-608

#3 mir 608

#4 mir-608

#5 rs4919510

#6 #1 OR #2 OR #3 OR #4 OR #5

#7 polymorphism

#8 variant

#9 mutation

#10 #7 OR #8 OR #9

#11 cancer

#12 tumor

#13 neoplasm

#14 #11 OR #12 OR #13

#15 #6 AND #10 AND #14

### Eligibility criteria

Studies were included based on the following criteria: (1) only case-control studies that focusing on the association between the microRNA-608 rs4919510 G>C polymorphism and DSCs risk; and (2) studies had to provided sufficient frequency data on the genotype distribution to evaluate the crude odds ratios (ORs) and 95% confidence intervals (CIs); and; (3) studies had to be published only in English and Chinese; and (4) only publications with the largest or most recently updated sample data were included when there were some overlapping or duplicate publications on the same theme.

### Data extraction and quality evaluation

Two researchers (Li and Song) independently reviewed and extracted relevant information from all included studies, including name of the first author, publishing date, country or region where the study was conducted, race, control design, sample sizes of the cases and controls, frequency data of genotype distribution, genotyping method, Hardy-Weinberg equilibrium (HWE) assessment in controls, and cancer type. Quality evaluation of the included studies was performed by the two researchers using the modified Newcastle-Ottawa scale (NOS). The scores ranged from 0 points (worst) to 11 points (best) (Table 4); studies with a score of 9 or higher were classified as high quality.

### Statistical analysis

We calculated ORs and 95% CIs to assess the association between microRNA-608 rs4919510 G>C polymorphism and DSCs risk. All pooled genetic models were examined, including the allele contrast (C vs. G), co-dominant models (GC vs. GG and CC vs. GG), dominant model (GC+CC vs. GG), and recessive model (CC vs. GG+GC). Heterogeneity between the included studies was calculated using Cochran's *Q*-test and I^2^ statistical method (Huedo-Medina et al., 2006). The fixed-effect model was used when the *I*^2^-value was less than 40%; otherwise, a random-effects model was used (Mantel and Haenszel, 1959; DerSimonian, 1996). Subgroup analyses were performed according to race (Asian, Caucasian, and African), control design (population base, hospital base), cancer type, subject number, and NOS evaluation. Moreover, meta-regression was performed to interpret the between-group heterogeneity. Cumulative meta-analyses were performed to assess the continuous tendency in the results. Furthermore, sensitivity analyses were conducted to examine the stability of the results by removing each study one by one. Potential publication biases were examined using Egger's linear regression and Begg's funnel plots. Statistical analyses were performed using STATA version 14.0 (Stata Corporation, College Station, TX, USA) (Begg and Mazumdar, 1994; Egger et al., 1997). A two-sided *P*-value less than 0.05 was considered statistically significant.

## Results

### Study characteristics

**Figure 1:**
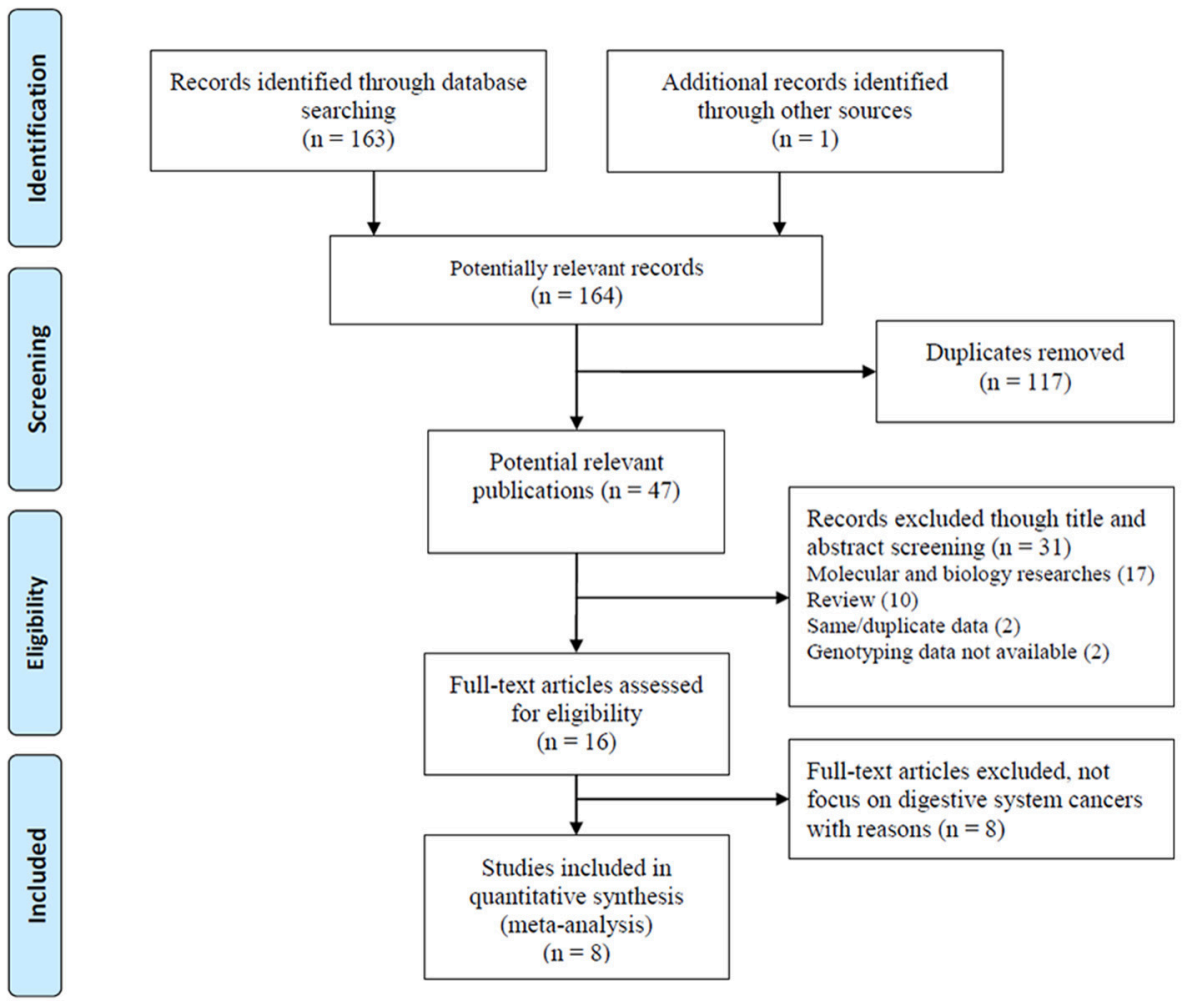
Flow diagram of the study selection process.

A total of 164 articles were collected. The study selection process was presented in Figure 1. One hundred and fifty-six articles were excluded during the comprehensive screening procedures based on the article titles, abstracts, and full texts. Thus, eight articles including nine independent case-control studies involving 5,224 patients and 5,612 controls were identified and included in this meta-analysis according to our inclusion criteria (Ryan et al., 2012; Zhang, 2012; Kupcinskas et al., 2014a,b; Wang et al., 2014; Zhang et al., 2015; Jiang et al., 2016; Ying et al., 2016). Five case-control studies included 4,430 cases and 4,403 controls in Asian populations(Zhang, 2012; Wang et al., 2014; Zhang et al., 2015; Jiang et al., 2016; Ying et al., 2016), thee case-control studies involved 700 cases and 1,024 controls in Caucasian populations (Ryan et al., 2012; Kupcinskas et al., 2014a,b), and one case-control study involved 94 cases and 185 controls in an African American population (Ryan et al., 2012). For genotyping, four studies used the Taqman method (Ryan et al., 2012; Kupcinskas et al., 2014a,b), three studies used the MassARRAY method (Wang et al., 2014; Jiang et al., 2016; Ying et al., 2016), and the remaining two studies used other methods (SNaPshot, PCR-RFLP) (Zhang, 2012; Zhang et al., 2015). Moreover, five studies focused on colorectal cancer(Ryan et al., 2012; Zhang, 2012; Kupcinskas et al., 2014a; Ying et al., 2016), two studies focused on gastric cancer (Kupcinskas et al., 2014b; Jiang et al., 2016), one study focused on hepatocellular cancer (Wang et al., 2014) and other focused on esophageal cancer (Zhang et al., 2015). The genotype distributions in all control groups were satisfied with the HWE. The characteristics of the included studies are presented in Table 1.

**Table 1:** Characteristics of included studies on MicroRNA-608 rs4919510 G>C polymorphisms and digestive system cancers risk.

							**Genotype distribution**										
							**Case**			**Control**							
**First author**	**Year**	**Country/Region**	**Racial**	**Source of controls**	**Case**	**Control**	**GG**	**GC**	**CC**	**GG**	**GC**	**CC**	**Genotyping methods**	***P* for HWE**	**MAF in control**	**Type**	**NOS**
Zhang MW	2012	China	Asian	PB	460	465	156	221	83	147	228	90	PCR-RFLP	0.92	0.44	CRC	9
Ryan BM-a	2012	USA	Caucasian	Mixed	145	248	7	48	90	8	71	169	Taqman	0.87	0.82	CRC	8
Ryan BM-b	2012	USA	African	Mixed	94	185	12	48	34	28	95	62	Taqman	0.39	0.59	CRC	8
Kupcinskas J-1	2014	Latvia	Caucasian	HB	363	350	25	88	250	13	86	251	Taqman	0.11	0.84	GC	8
Kupcinskas J-2	2014	Latvia	Caucasian	HB	192	426	7	47	138	12	96	318	Taqman	0.16	0.86	CRC	7
Wang R	2014	China	Asian	PB	993	992	304	500	189	318	497	177	MassARRAY	0.48	0.43	HC	8
Zhang P	2015	China	Asian	PB	738	882	217	384	137	291	440	151	SNaPshot	0.48	0.42	ESCC	9
Jiang J	2016	China	Asian	HB	894	989	278	451	165	296	483	210	MassARRAY	0.62	0.46	GC	9
Ying HQ	2016	China	Asian	HB	1,345	1,075	232	690	423	250	512	313	MassARRAY	0.15	0.53	CRC	8

*HWE in control*.

*CRC, Colorectal cancer; GC, Gastric cancer; HC, Hepatocellular cancer; ESCC, Esophageal squamous cell carcinoma; MAF, Minor allele frequency in control group; PB, Population-based; HB, Hospital-based; Mixed, combined PB with HB*.

### Meta-analysis

**Figure 2:**
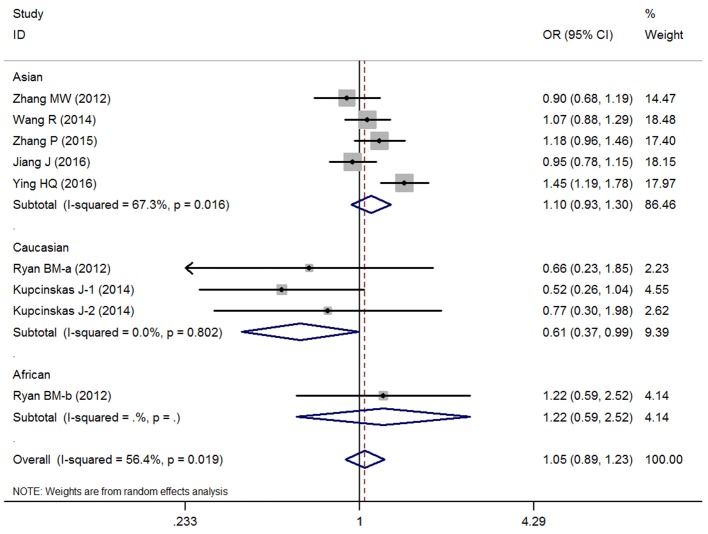
OR and 95% CIs of the associations between microRNA-608 rs4919510 G>C polymorphism and digestive system cancer risk in GC+CC vs. GG model.

Overall, no significant association between the microRNA-608 rs4919510 G>C polymorphism and DSCs risk was observed with the included studies (C vs. G: OR = 1.00, 95% CI = 0.91–1.10, *P* = 0.98, *I*^2^ = 53.4%; GC vs. GG: OR = 1.07, 95% CI = 0.93–1.24, *P* = 0.35, *I*^2^ = 43.0%; CC vs. GG: OR = 1.01, 95% CI = 0.83–1.24, *P* = 0.90, *I*^2^ = 56.4%; GC+CC vs. GG: OR = 1.05, 95% CI = 0.89–1.23, *P* = 0.58, *I*^2^ = 56.4%, (Figure 2); CC vs. GG+GC: OR = 0.99, 95% CI = 0.91–1.09, *P* = 0.89, *I*^2^ = 0%) (Supplementary Figure S1).

### Subgroup and meta-regression analysis

The results of race diversity demonstrated some significant protective effects between microRNA-608 rs4919510 G>C polymorphism and DSCs risk in Caucasian populations (C vs. G: OR = 0.82, 95% CI = 0.68–0.99, *P* = 0.03, *I*^2^ = 0%; CC vs. GG: OR = 0.59, 95% CI = 0.36–0.97, *P* = 0.04, *I*^2^ = 0%; GC+CC vs. GG: OR = 0.61, 95% CI = 0.37–0.99, *P* = 0.05, *I*^2^ = 0%, Figure 2). But the other Subgroup analysis based on control design, cancer type, subject number, and NOS evaluation did not present any significant association between microRNA-608 rs4919510 G>C polymorphism and DSCs risk. All these statistical results were presented in Table 2. Moreover, meta-regression were conducted due to the slight heterogeneity between included studies existed, but the statistical test did not find any remarkable factors that contributed to the current heterogeneity (Table 3).

**Table 2:** Summary ORs and 95% CI of MicroRNA-608 rs4919510 G>C polymorphisms and digestive system cancers risk.

	N*	**C vs. G**				**GC vs. GG**				**CC vs. GG**				**GC**+**CC vs. GG**				**CC vs. GG**+**GC**			
		**OR**	**95% CI**	***P*^a^**	***I^2^*(%)**	**OR**	**95% CI**	***P*^a^**	***I^2^*(%)**	**OR**	**95% CI**	***P*^a^**	***I^2^*(%)**	**OR**	**95% CI**	***P*^a^**	***I^2^*(%)**	**OR**	**95% CI**	***P*^a^**	***I^2^*(%)**
Total	9	1.00	0.91–1.10	0.98	53.4	1.07	0.93-1.24	0.35	43.0	1.01	0.83–1.24	0.90	56.4	1.05	0.89–1.23	0.58	56.4	0.99	0.91–1.09	0.89	0
**RACIAL**																					
Asian	5	1.04	0.95–1.15	0.40	61.3	1.11	0.94–1.30	0.18	57.5	1.09	0.88–1.35	0.43	66.2	1.10	0.93–1.30	0.25	67.3	1.03	0.93–1.14	0.62	17.6
Caucasian	3	0.82	0.68–0.99	0.03	0	0.65	0.39–1.09	0.10	0	0.59	0.36–0.97	0.04	0	0.61	0.37–0.99	0.05	0	0.84	0.68–1.04	0.12	0
African	1	1.11	0.78–1.59	0.56	NA	1.18	0.55–2.52	0.67	NA	1.28	0.58–2.83	0.54	NA	1.22	0.59–2.52	0.59	NA	1.12	0.67–1.89	0.66	NA
**DESIGN**																					
PB	3	1.05	0.96–1.14	0.29	11.8	1.06	0.93–1.21	0.38	0	1.09	0.92–1.30	0.32	0	1.07	0.94–1.21	0.30	15.3	1.05	0.91–1.22	0.50	0
HB	4	0.96	0.80–1.16	0.70	75.8	1.02	0.71–1.47	0.91	73.1	0.90	0.57–1.42	0.65	80.0	0.97	0.66–1.43	0.87	79.6	0.97	0.86–1.10	0.63	37.3
Mixed	2	0.93	0.66–1.32	0.69	44.6	1.02	0.55–1.91	0.94	0	0.96	0.47–1.95	0.90	18.7	1.00	0.55–1.81	1.00	0	0.89	0.64–1.25	0.51	20.3
**TYPE**																					
CRC	5	1.00	0.84–1.18	0.96	58.6	1.11	0.83–1.50	0.48	47.4	1.06	0.75–1.50	0.74	51.0 .0	1.07	0.77–1.49	0.67	59.5	1.02	0.89–1.16	0.82	0
GC	2	0.90	0.80–1.01	0.08	0	0.81	0.45–1.44	0.47	61.3	0.79	0.62–1.00	0.05	37.9	0.77	0.44–1.34	0.36	62.6	0.85	0.71–1.02	0.09	0
Other	2	1.08	0.98–1.18	0.11	0	1.10–	0.95–1.28	0.20	0	1.16	0.96–1.41	0.13	0	1.12	0.97–1.23	0.12	0	1.10	0.921.29	0.31	0
**SUBJECTS**																					
< 1,000	5	0.90	0.79–1.01	0.08	0	0.87	0.69–1.11	0.27	0	0.81	0.61–1.07	0.13	0	0.85	0.58–1.07	0.17	0	0.89	0.75–1.05	0.17	0
>1,000	4	1.06	0.96–1.18	0.24	64.0	1.15	0.98–1.36	0.09	58.9	1.14	0.90–1.44	0.28	69.7	1.15	0.96–1.37	0.13	69.0	1.04	0.93–1.16	0.49	31.3
**NOS EVALUATION**																					
NOS ≥ 9	3	0.99	0.87–1.12.	0.83	51.0	1.04	0.91–1.19	0.60	0.9	0.95	0.75–1.23	0.77	48.6	1.01	0.93–1.15	0.81	37.6	0.94	0.81–1.09	0.43	19.5
NOS < 9	6	1.00	0.87–1.15	0.78	55.9	1.07	0.82–1.41	0.63	52.4	1.03	0.76–1.41	0.84	55.6	1.04	0.78–1.38	0.80	60.5	1.02	0.91–1.15	0.68	0

* *Numbers of comparisons*.

a *P-value for OR evaluation*.

**Table 3:** Characteristics of included covariate in meta-regression analysis in all five genetic models.

	**C vs. G**		**GC vs. GG**		**CC vs. GG**		**GC**+**CC vs. GG**		**CC vs. GG**+**GC**	
	***t***	***P***	***t***	***P***	***t***	***P***	***t***	***P***	***t***	***P***
Racial	−1.03	0.337	−0.90	0.400	−0.85	0.425	−0.92	0.388	−0.86	0.417
Design	−0.71	0.501	0.08	0.935	−0.46	0.661	−0.20	0.845	−0.99	0.354
Type	0.48	0.648	−0.14	0.891	0.21	0.842	0.05	0.960	0.56	0.594
Subjects	2.01	0.084	1.76	0.122	1.75	0.123	1.86	0.105	1.48	0.184
NOS evaluation	0.21	0.841	0.47	0.656	0.39	0.711	0.33	0.748	0.68	0.520

**Table 4:** Scale for quality evaluation.

**Criteria**	**Score**
**REPRESENTATIVENESS OF CASES**	
Consecutive/randomly selected cases with clearly defined sampling frame	2
Not consecutive/randomly selected case or without clearly defined sampling frame	1
Not described	0
**SOURCE OF CONTROLS**	
Population- or Healthy-based	2
Hospital-bases	1
Not described	0
**HARDY-WEINBERG EQUILIBRIUM IN CONTROLS**	
Hardy-Weinberg equilibrium	2
Hardy-Weinberg disequilibrium	1
Not available	0
**GENOTYPING EXAMINATION**	
Genotyping done under “blinded” condition and repeated again	2
Genotyping done under “blinded” condition or repeated again	1
Unblinded done or not mentioned and unrepeated	0
**SUBJECTS**	
Number >1,000	1
Number < 1,000	0
**ASSOCIATION ASSESSMENT**	
Assess association between genotypes and cancer with appropriate statistics and adjustment for confounders	2
Assess association between genotypes and cancer with appropriate statistics and without adjustment for confounders	1
Inappropriate statistics used	0

### Cumulative and sensitivity analyses

**Figure 3:**
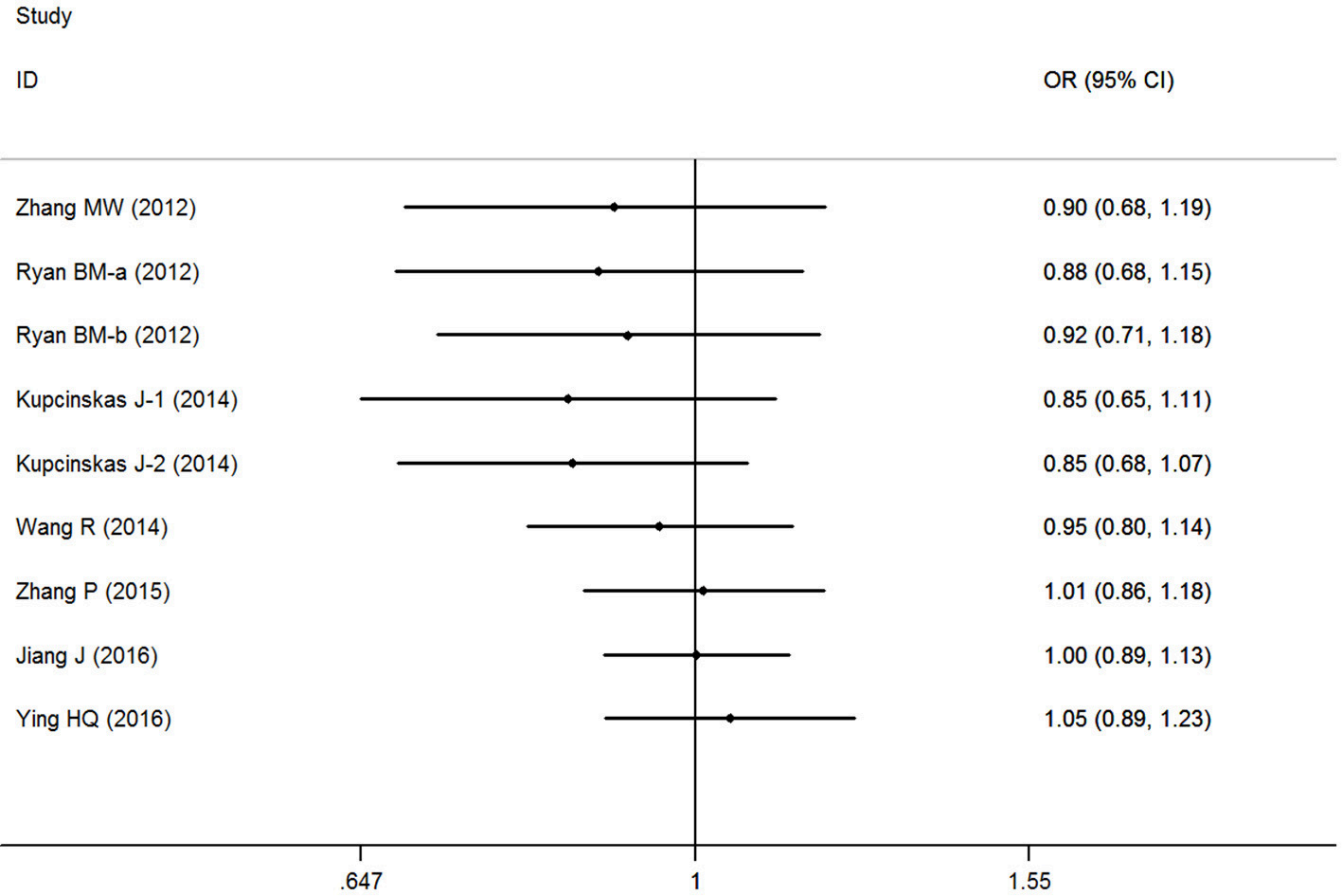
Cumulative meta-analyses according to publication year in GC+CC vs. GG model of microRNA-608 rs4919510 G>C polymorphism.

**Figure 4:**
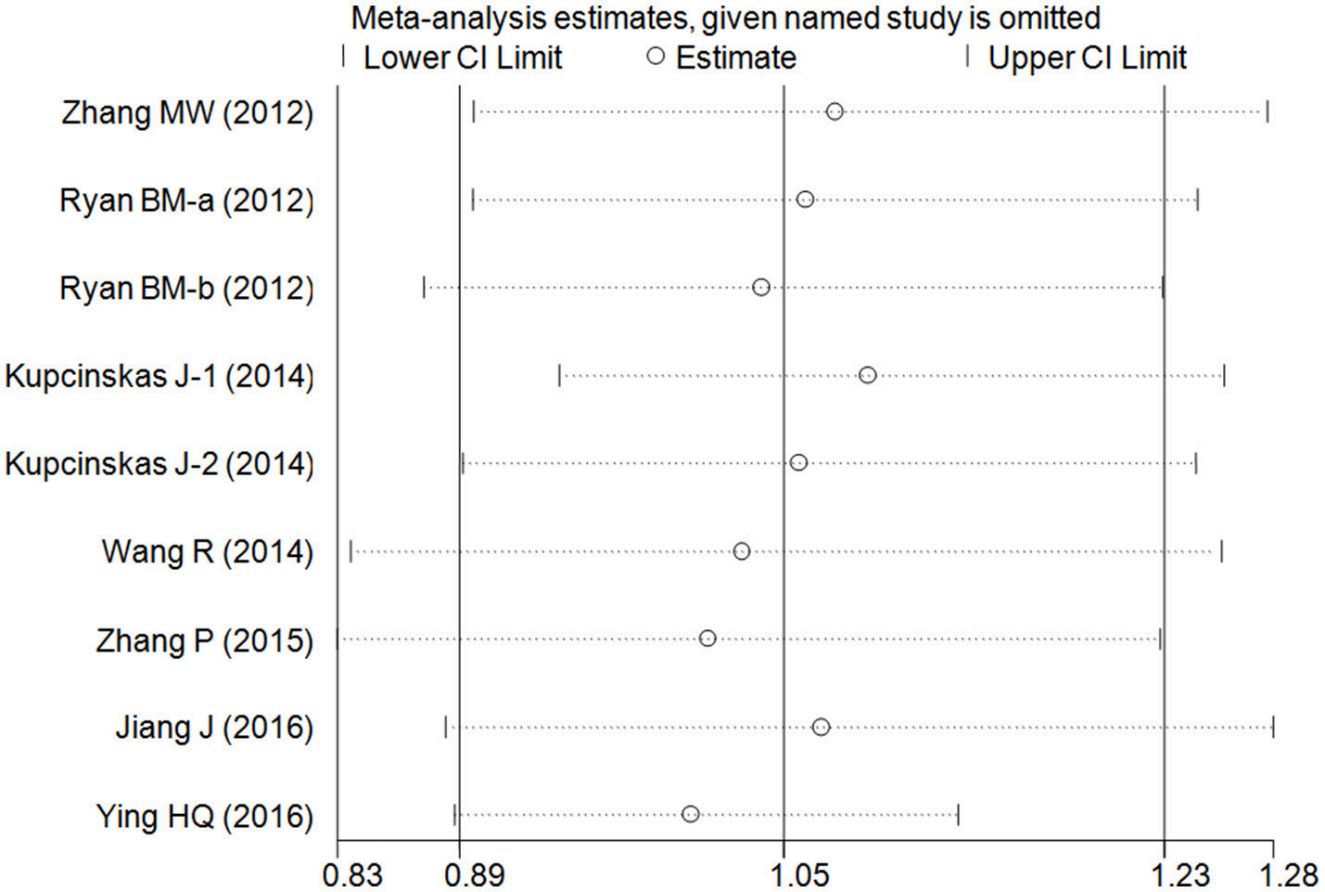
Sensitivity analysis involving deletion of each study to reflect the influence of the individual dataset to the pooled ORs in GC+CC vs. GG model of microRNA-608 rs4919510 G>C polymorphism.

Cumulative analysis indicated a consistent tendency accompanied with the continuously published studies (Figure 3 for GC+CC vs. GG model) (Supplementary Figure S2). Sensitive analyses were performed by omitting each study one at a time, and the results did not indicate any significant changes (Figure 4 for GC+CC vs. GG model) (Supplementary Figure S3).

### Publication bias

**Figure 5:**
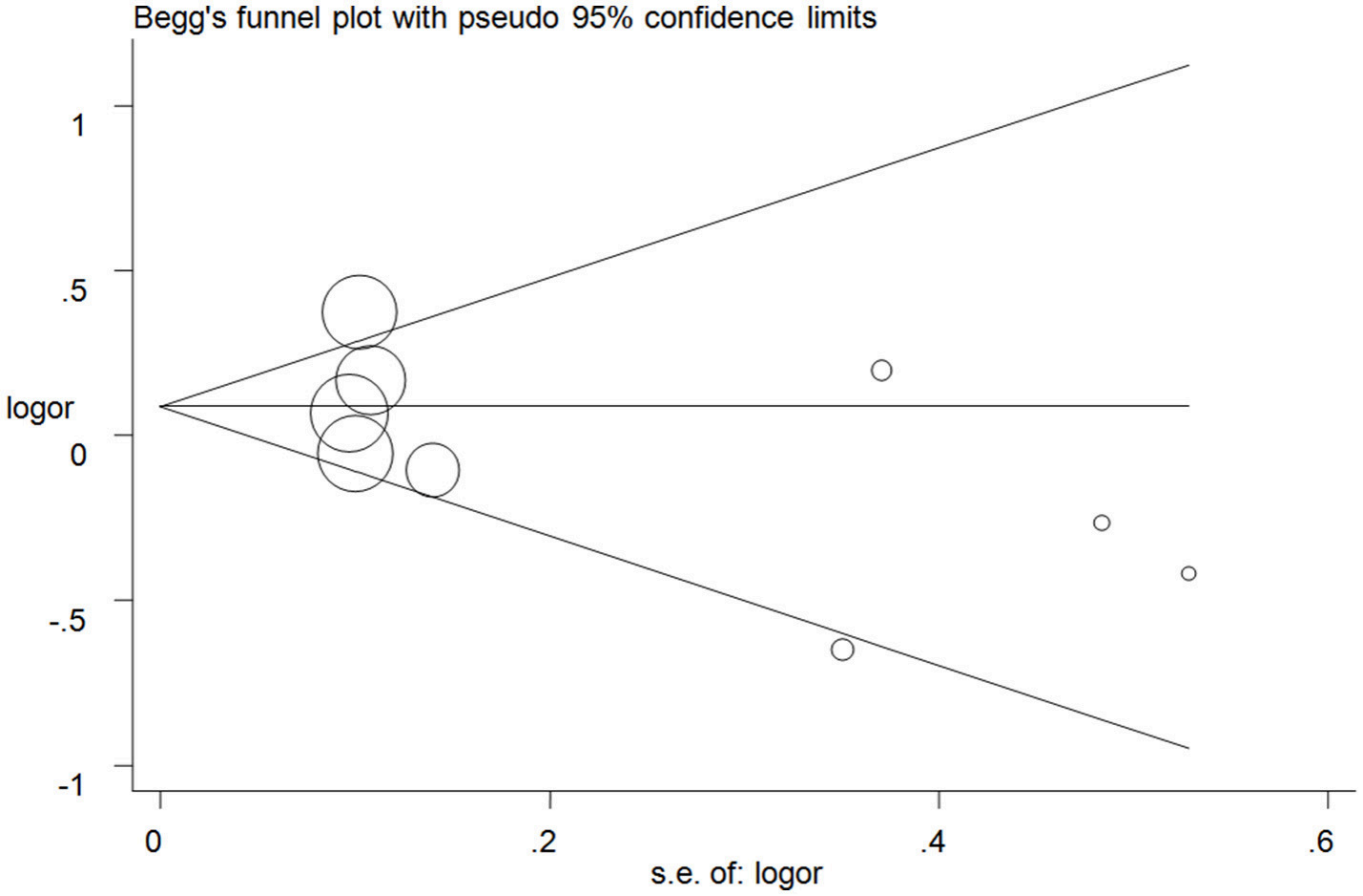
Funnel plot analysis to detect publication bias for GC+CC vs. GG model of microRNA-608 rs4919510 G>C polymorphism. Circles represent the weight of the studies.

Publication bias was examined with Begg's test and no apparent asymmetry of the funnel plot was found (Figure 5 for GC+CC vs. GG model) (Supplementary Figure S4). These results were confirmed with Egger's test (C vs. G: *P* = 0.11; GC vs. GG: *P* = 0.24; CC vs. GG: *P* = 0.16; GC+CC vs. GG: *P* = 0.22; CC vs. GG+GC: *P* = 0.19).

## Discussion

MicroRNAs is a family of small, noncoding, evolutionarily conserved endogenous RNAs consisting of 22 nucleotides that can regulate gene expression through a post-transcriptional pathway binding to the 3′-UTR of target mRNAs (Lagos-Quintana et al., 2001). MicroRNAs take part in numerous biologic processes, including cell proliferation, differentiation and apoptosis (Ambros, 2003). Evidences have shown that microRNAs play a composite function for oncogenes and tumor suppressor genes. Some mutations, such as SNPs in microRNAs or their binding site, may alter cancer susceptibility in various tumor types and different populations (Niu et al., 2014, 2015).

MicroRNA-608 is novel small RNA that has been shown to be significantly associated with cancer development, metastasis and recurrence. Wang et al. had found that the expression of miR-608 expression was significantly reduced in hepatocellular carcinoma and presented a negative correlation of the tumor size, differentiation, and so on (Wang K. et al., 2016). Yang et al. reported that the miR-608 could suppress the tumorigenesis of colon cancer cells by targeting NAA10 mRNA for degradation. And the overexpression of miR-608 could decrease NAA10 mRNA and protein levels, and thereby suppressed cell proliferation, migration, and cell-cycle progression (Yang et al., 2016).

Rs4919510 G>C is the most common SNP locus in microRNA-608 and might contribute a different free energy to its binding site of target genes, including INSR,CD4, GHR and RXRB (Landi et al., 2008). According the research of Huang et al., the variant genotypes (CG/GG) and variant G-allele were specifically associated with increased risk of HER2-positive subtype in breast cancer patients. HSF1 is a predicted target of miR-608 and the C-to-G substitution of rs4919510 might weaken the suppression of HSF1 mRNA by miR-608, leading to relatively high expression of HSF1 protein, which could up-regulate the expression of HER2 and facilitated tumorigenesis. The above evidence indicated that the alteration of free energy caused by gene mutation between miR-608 and targeted mRNA would influence the protein expression and change the cancer susceptibility (Huang et al., 2012). Since 2008, a series of case-control studies focused on microRNA-608 rs4919510 G>C polymorphism and DSCs risk were published, but the conclusions were inconsistent. Kupcinskas et al. explored the association between microRNA-608 rs4919510 G>C polymorphism and gastric cancer and found an apparently increased risk of gastric cancer in a Latvian population with GG genotype (GG vs. CC, OR = 2.34, 95% CI, 1.08–5.04) (Kupcinskas et al., 2014b). Conversely, Ying et al. evaluated the microRNA-608 rs4919510 G>C polymorphism in Chinese individuals and concluded that the rs4919510 G-allele and genotype GG were protective factors for stage 0 to II colorectal cancer (CRC) (GG vs. CC, OR = 0.70, 95% CI = 0.55–0.88) (Ying et al., 2016). However, other studies did not find any significant relationship between the microRNA-608 rs4919510 G>C polymorphism and DSCs risk, including these studies by Wang et al. (2014) and Zhang et al. (2015). How can we conduct a more precise assessment of the association between microRNA-608 rs4919510 G>C polymorphism and DSC risk with these inconsistent results? As we know, few studies and small sample size maybe the important reasons due to the confusing results. Therefore, we conducted the meta-analysis with nine published case-control studies to investigate the association between the microRNA-608 rs4919510 G>C polymorphism and DSCs susceptibility. Overall, our meta-analysis did not identify any significant association in all of the genetic models. To our knowledge, the potential real results might be confounded or even conceal by some subgroup factors, such as race diversity, control design, cancer type, subject number, and NOS evaluation. So, we subsequently conducted stratified analyses by these subgroup factors. The results indicate that the microRNA-608 rs4919510 G>C polymorphism play an important protective effect against DSCs development in Caucasian population only, but not in other subgroup analysis. It was supposed that the racial diversity maybe the critical factor that contributed to the different result. However, according to current results in general population and subgroups, the precise biological mechanism of the association between microRNA-608 rs4919510 G>C polymorphism and DSCs risk were remains unclear. To our minds, the possible explanation was that the microRNA-608 rs4919510 G>C polymorphism not participated in cancer susceptibility directly, but play a connected and synergism role during the development of cancer. All above results and speculations were needed to confirm with new molecular and epidemiological studies in various races.

In 2017, Liu et al. (2016) and Wu et al. (2017) published two meta-analysis assessing the association between the microRNA-608 rs4919510 G>C polymorphism and cancer risk. Their researches included 10 and 18 case-control studies respectively, which consisted of thyroid cancer, breast cancer, lung cancer and others. Liu et al. indicated that the presence of CC genotypes might be protective against tumorigenesis, especially in Caucasians. And Wu et al. suggested that CG genotypes would increase the cancer risk in the Chinese populations. However, the results of their meta-analysis focused on the general cancer, and the association between the microRNA-608 rs4919510 G>C polymorphism and DSCs risk was not conducted. To our knowledge, this is the first meta-analysis to explore the association between the microRNA-608 rs4919510 G>C polymorphism and DSCs risk. Compared with previous meta-analyses, the present meta-analysis used a more scientific retrieval strategy and a larger sample size. Additionally, more rigorous methodology, including cumulative and sensitivity analyses and quality evaluation with modified NOS, were used to estimate the genetic effects of the microRNA-608 rs4919510 G>C polymorphism on carcinogenesis. This provided a more accurate evaluation of the association between the microRNA-608 rs4919510 G>C polymorphism and DSCs risk.

However, some limitations of this analysis could not be avoided and should be addressed. First, some heterogeneity was apparent among the included studies, which might distort the results in the current meta-analysis. The meta-regression was conducted but not found any distinct factor that contributes to the current heterogeneity. Fortunately, the heterogeneity was partly alleviated in the subsequent stratified analysis, such as race diversity and cancer type. Second, this meta-analysis only focused on one SNP locus (microRNA-608 rs4919510 G>C), and the results were calculated without gene-gene and gene-environment risk factors, leading to a failure to interpret the potential interaction mechanisms. Third, almost all of the included studies focused on Asian and Caucasian populations, which would restrict the application of our results to other populations.

In summary, our meta-analysis demonstrated that the microRNA-608 rs4919510 G>C polymorphism might play an important role in DSCs susceptibility. Further studies in different races and regions with larger population sizes are needed to confirm our findings.

## Author contributions

X-FL, J-KS, and Y-MN conceived the study. X-FL, J-KS, and J-WC searched the databases and extracted the data. J-KS, Y-QZ, and ML analyzed the data. X-FL, J-KS, and J-WC wrote the draft of the paper. JZ and Y-MN reviewed the manuscript. All the authors approved the final manuscript.

### Conflict of interest statement

The authors declare that the research was conducted in the absence of any commercial or financial relationships that could be construed as a potential conflict of interest.
