# Supplementary material for: No Association Between MicroRNA-608 rs4919510 G>C Polymorphism and Digestive System Cancers Susceptibility: A Meta-Analysis Based on 10,836 Individuals
Source: Front Physiol. 2018 Jun 7;9:705. doi: 10.3389/fphys.2018.00705 (PMC5999779; doi:10.3389/fphys.2018.00705)
Supplement: Supplementary file 1 [file Presentation_1.PDF]

Supplementary Figure Legends

Supplementary Figure S1. OR and 95% CIs of the associations between microRNA-608 rs4919510 G>C polymorphism and digestive system cancer risk (A for C vs. G model; B for GC vs. GG model; C for CC vs. GG model; D for CC vs. GG+GC model).

Supplementary Figure S2. Cumulative meta-analyses according to publication year in microRNA-608 rs4919510 G>C polymorphism and digestive system cancer risk (A for C vs. G model; B for GC vs. GG model; C for CC vs. GG model; D for CC vs. GG+GC model).

Supplementary Figure S3 Sensitivity analysis through deleting each study to reflect the influence of the individual dataset to the pooled ORs in microRNA-608 rs4919510 G>C polymorphism and digestive system cancer risk (A for C vs. G model; B for GC vs. GG model; C for CC vs. GG model; D for CC vs. GG+GC model).

Supplementary Figure S4. Funnel plot analysis to detect publication bias in microRNA-608 rs4919510 G>C polymorphism (A for C vs. G model; B for GC vs. GG model; C for CC vs. GG model; D for CC vs. GG+GC model). Circles represent the weight of the studies.
