# Supplementary figures and images for: No Association Between MicroRNA-608 rs4919510 G>C Polymorphism and Digestive System Cancers Susceptibility: A Meta-Analysis Based on 10,836 Individuals
Source: Front Physiol. 2018 Jun 7;9:705. doi: 10.3389/fphys.2018.00705 (PMC5999779; doi:10.3389/fphys.2018.00705)

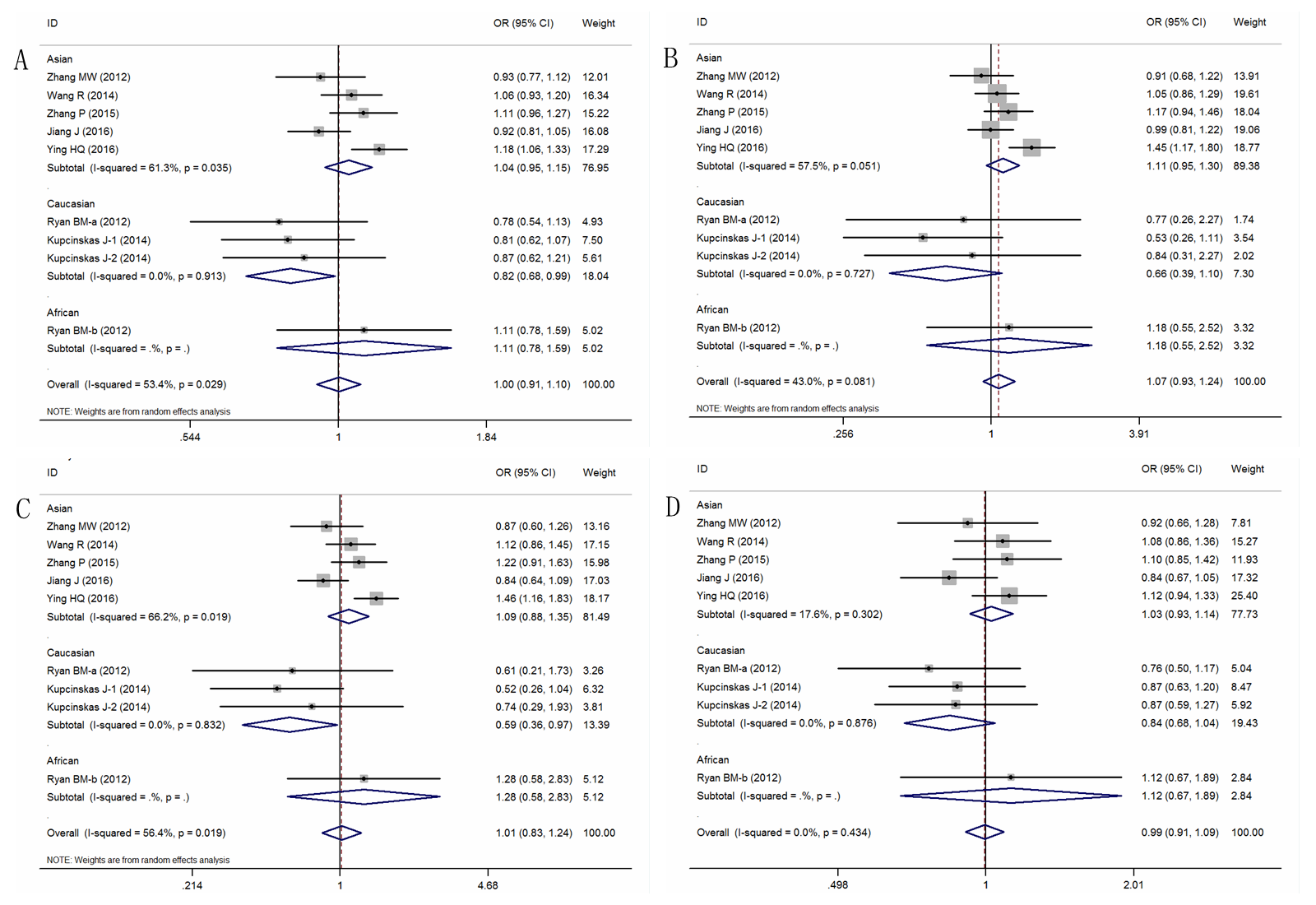

Supplement: Supplementary file 2 [file Image_1.TIFF]

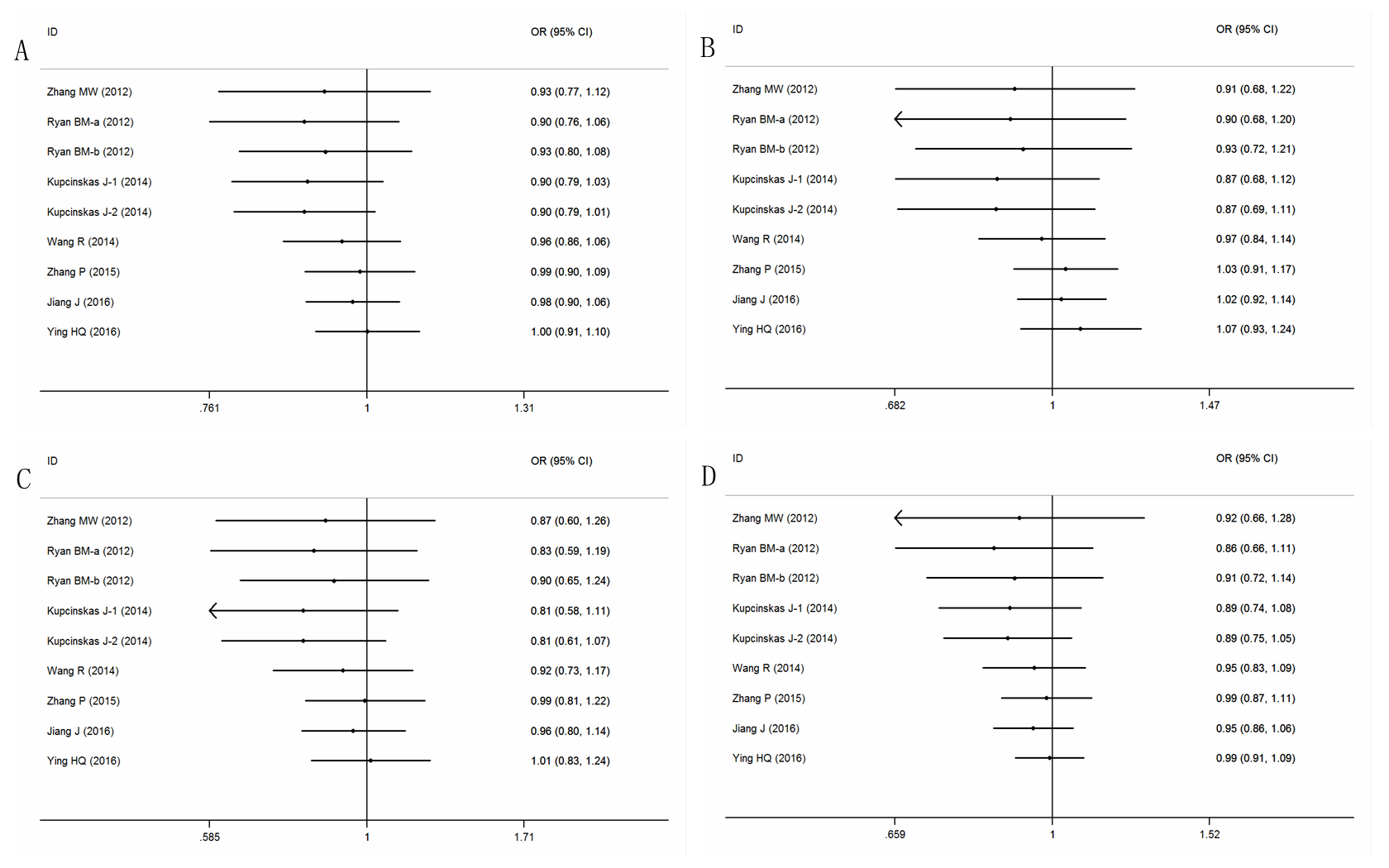

Supplement: Supplementary file 3 [file Image_2.TIFF]

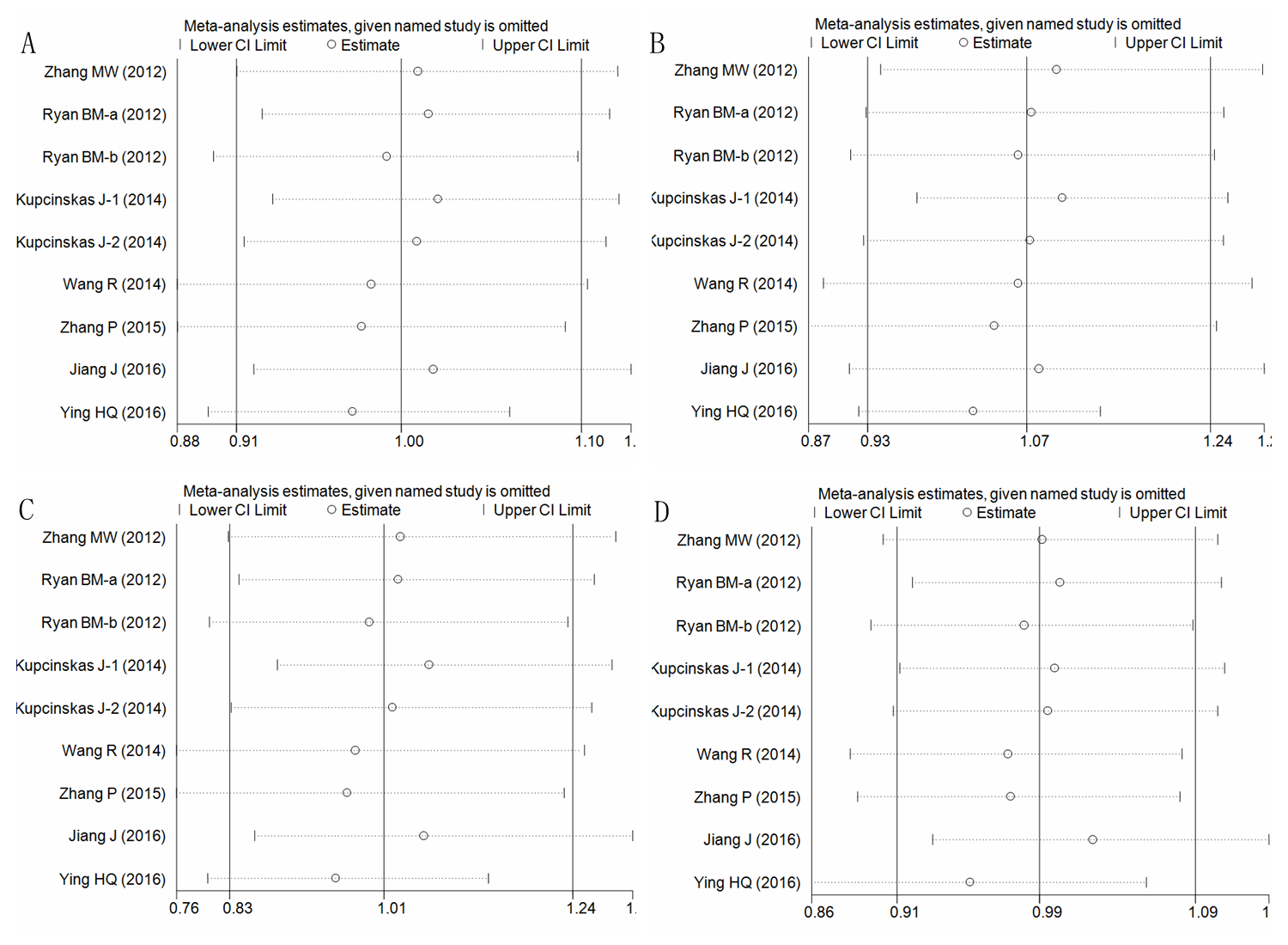

Supplement: Supplementary file 4 [file Image_3.TIFF]

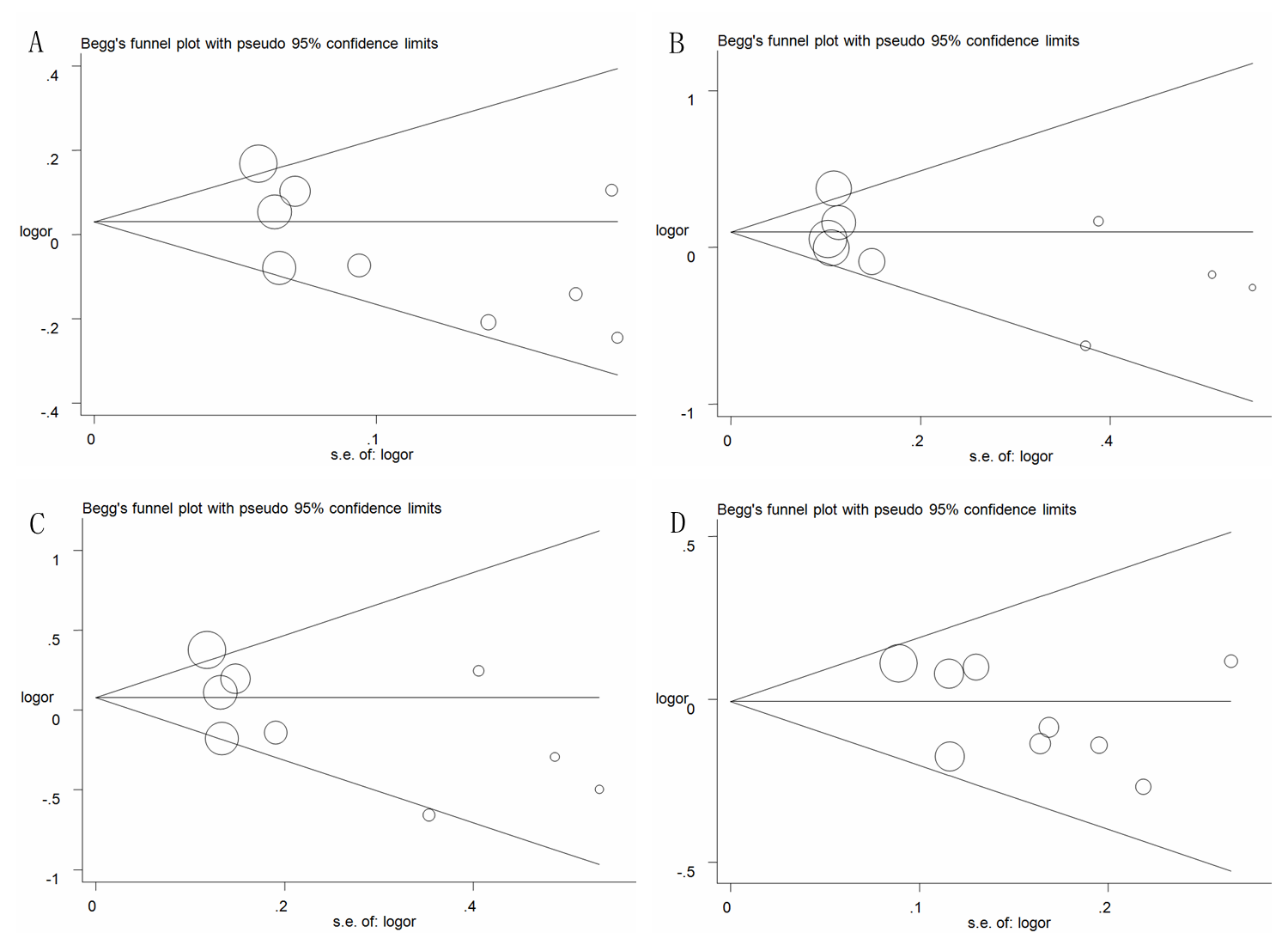

Supplement: Supplementary file 5 [file Image_4.TIFF]
